# Cooperative interaction of interferon regulatory factor -1 and bromodomain—containing protein 4 on RNA polymerase activation for intrinsic innate immunity

**DOI:** 10.3389/fimmu.2024.1366235

**Published:** 2024-03-27

**Authors:** Xiaofang Xu, Dianhua Qiao, Allan R. Brasier

**Affiliations:** ^1^ Department of Medicine, University of Wisconsin-Madison School of Medicine and Public Health (SMPH), Madison, WI, United States; ^2^ Institute for Clinical and Translational Research, University of Wisconsin-Madison, Madison, WI, United States

**Keywords:** RSV, transcriptional elongation, RNA pol II, small airway basal cells, multipotent stem cell

## Abstract

**Introduction:**

The human orthopneumovirus, Respiratory Syncytial Virus (RSV), is the causative agent of severe lower respiratory tract infections (LRTI) and exacerbations of chronic lung diseases. In immune competent hosts, RSV productively infects highly differentiated epithelial cells, where it elicits robust anti-viral, cytokine and remodeling programs. By contrast, basal cells are relatively resistant to RSV infection, in part, because of constitutive expression of an intrinsic innate immune response (IIR) consisting of a subgroup of interferon (IFN) responsive genes. The mechanisms controlling the intrinsic IIR are not known.

**Methods:**

Here, we use human small airway epithelial cell hSAECs as a multipotent airway stem cell model to examine regulatory control of an intrinsic IIR pathway.

**Results:**

We find hSAECs express patterns of intrinsic IIRs, highly conserved with pluri- and multi-potent stem cells. We demonstrate a core intrinsic IIR network consisting of Bone Marrow Stromal Cell Antigen 2 (*Bst2*), Interferon Induced Transmembrane Protein 1 (*IFITM1*) and Toll-like receptor (*TLR3*) expression are directly under IRF1 control. Moreover, expression of this intrinsic core is rate-limited by ambient IRF1• phospho-Ser 2 CTD RNA Polymerase II (pSer2 Pol II) complexes binding to their proximal promoters. In response to RSV infection, the abundance of IRF1 and pSer2 Pol II binding is dramatically increased, with IRF1 complexing to the BRD4 chromatin remodeling complex (CRC). Using chromatin immunoprecipitation in IRF1 KD cells, we find that the binding of BRD4 is IRF1 independent. Using a small molecule inhibitor of the BRD4 acetyl lysine binding bromodomain (BRD4i), we further find that BRD4 bromodomain interactions are required for stable BRD4 promoter binding to the intrinsic IIR core promoters, as well as for RSV-inducible pSer2 Pol II recruitment. Surprisingly, BRD4i does not disrupt IRF1-BRD4 interactions, but disrupts both RSV-induced BRD4 and IRF1 interactions with pSer2 Pol II.

**Conclusions:**

We conclude that the IRF1 functions in two modes- in absence of infection, ambient IRF1 mediates constitutive expression of the intrinsic IIR, whereas in response to RSV infection, the BRD4 CRC independently activates pSer2 Pol II to mediates robust expression of the intrinsic IIR. These data provide insight into molecular control of anti-viral defenses of airway basal cells.

## 1 Introduction

RSV is a major etiologic pathogen for infant lower respiratory tract infection (LRTI) and recurrent infection producing substantial morbidity throughout life (1). In children less than five years of age, RSV is the most common cause of hospitalization in the US (2) and, globally, responsible for 1/3 of severe infections (lower respiratory tract infections; LRTIs) (3). Although mortality is rare in Western countries, severe RSV LRTIs exert substantial long-term morbidity. RSV LRTIs are linked to recurrent wheezing, allergic sensitization, persistent reductions in lung function and enhanced utilization of health care resources (1, 4–6). Recently, a large observational cohort study concluded that severe RSV LRTI produced a 2-fold increased risk of premature death from respiratory disease (5). Improved understanding of the mechanisms and consequences of disease are needed.

Understanding how RSV replication is restricted in the epithelium is important for disease outcomes because the magnitude of the initial RSV replication determines disease severity, inflammation and mucus production in humans (7). In natural infections of immune-competent individuals, RSV primarily replicates in the differentiated epithelial surface (8–10). Studies of naturally acquired (11) and experimentally induced (12) RSV infections have found that RSV replicates in small bronchiolar epithelial cells. Systems level studies of small airway cells have shown that this population is primed to produce greater amounts of neutrophilic-, T helper 2 (Th2)-polarizing-, mucogenic cytokines and mesenchymal growth factors (13, 14). Intracellularly, RSV replicates in Endoplasmic Reticulum (ER)-associated stress granules composed of RSV RNA, RNA-binding proteins and translationally stalled cellular mRNAs. These entities activate pattern recognition receptors (PRRs) retinoic acid inducible gene-I (RIGI) and Toll-like receptor 3 (TLR3) (15–17) that form active TANK-Binding Kinase and IκB Kinase (IKK) signalsomes, stimulating interferon regulatory factor (IRF1), activating protein 1 (AP1) and NF-κB (17–19). More recent work has shown that RSV is also a potent activator of the unfolded protein response that results in inducible phenotypic changes known as “epithelial plasticity” and upregulation of N-glycosylation pathways controlling extracellular matrix production and mucin secretion (20, 21). The exuberant, innate response of small airways epithelium explains the observations that, in fatal cases of RSV LRTI, acute hypoxic respiratory failure is associated with mucus plugging and ventilation-perfusion mismatch in the small airways (11).

The findings that the airway epithelial surface exhibits regionally distinct responses to RSV infection are functionally and pathologically important. This regional epithelial specialization is maintained by distinct populations of progenitor cells that repopulate the surface in response to injury or infection. In the small airway, basal cells constitute 6-30% of the total cellular population that facilitate regeneration of the epithelial surface after injury (22). Importantly, lineage mapping studies in mice have found that tumor protein 63 (TP63)- and keratin 5 (KRT5)-expressing progenitors from the bronchiolar epithelium repopulate the distal airway and type I and II alveolar cells after viral injury (23, 24).

It has been widely observed that pluripotent and multipotent tissue stem cells are resistant to a variety of RNA virus infections through constitutive expression of a gene network termed the “intrinsic” innate immune response [IIR; (25)]. Here, stem cell precursors constitutively express a core of ~100 IFN-Stimulated Genes (ISGs), including the Bone Marrow Stromal Cell Antigen 2 (*Bst2*), Interferon Induced Transmembrane Protein 1 (*IFITM1*) and Toll-like receptor (*TLR3*) whose expression provide a constitutive anti-viral defense. The intrinsic innate response genes are expressed in a manner independent of IFN activity, and their expression decline as the cells fully differentiate, whereupon the inducible expression of IFNs become the major anti-viral pathways used (25).

Mechanistic studies have shown that RSV replication in differentiated epithelial cells activate both the cytoplasmic pattern recognition receptor, RIG-I (17), as well as the membrane Toll like receptor-3 (TLR3) that converge on the IκB kinase (IKK) signalsome. The IKK signalsome, in turn, activates an NFκB-IRF cross-talk pathway that produces the genomic program of the IIR (26). The initial phase of the IIR is mediated by NFκB and IRF3, cytoplasmic sequestered transcription factors that translocate into the nucleus and trigger expression of ISGs and cytokines, resulting in apoptosis and cellular plasticity (26–28). Downstream, the ISGs IRF-1 and -7 are highly induced by NFκB which serve to sustain the anti-viral response. Because of its central role in the IIR, the mechanism by which NFκB regulates immediate early genes has been studied in detail (29, 30). Activated NFκB undergoes a coupled phosphorylation-acetylation reaction to bind to Bromodomain containing protein 4 (BRD4) within the positive transcription elongation factor complex (31, 32). Our studies have shown that BRD4 is required for cyclin-dependent kinase 9 (CDK9) recruitment and phospho-Ser 2 carboxy-terminal domain (CTD) RNA polymerase (Pol) II formation on the promoters of *IRF1*, *IRF7*, and *RIG-I*, producing their enhanced expression by transcriptional elongation (29). Although this mechanism applies to highly differentiated epithelial cells, the mechanisms how the intrinsic IIR is activated in airway multipotent basal cells is not fully explored.

Investigations of stratified mucociliary epithelial models have discovered that RSV replicates in ciliated cells and differentiated small airway cells. By contrast, basal epithelial cells are relatively resistant to RSV infection, requiring disruption of the overlying ciliated cells in pseudostratified monolayers (33). To better understand the anti-viral response of airway basal cells, we investigate the presence and regulation of intrinsic immunity in TP63^+^/KRT5^+^ positive basal cells from the lower airways. Here we observe that hSAECs express the core components of the intrinsic IIR pathway and investigate the mechanistic role of IRF1 in their expression. We observe IRF1 is constitutively engaged with activated RNA Pol II, controlling the intrinsic response. We find that IRF1 forms dynamic complexes with BRD4 in response to innate activation RSV infection. The actions of IRF1 and BRD4 are both required for stable accumulation of pSer2 RNA Pol II on IRF1-dependent ISGs. These data indicate that the cooperative actions of IRF1 and the BRD4 to maintain pSer2 Pol II is required for maintenance of intrinsic immunity in airway basal cells.

## 2 Materials and methods

### 2.1 Cell cultures and siRNA transfection

Telomerase-immortalized primary human small airway epithelial (hSAECs) were grown in small airway epithelial cell growth medium (SAGM, Lonza, Walkersville, MD, USA) in 5% CO_2_. For air-liquid interface (ALI) culture, cells were grown on transwell inserts (Corning 3460) in PneumaCult-ALI media (STEMCELL05001). Cells were harvested 21 days later. Sucrose cushion-purified RSV Long strain was prepared (34). The BRD4 inhibitor ZL0454 was synthesized and HPLC-purified (35, 36). ZL0454 was used at 10 μM concentration in cell culture medium and was added at the same time cells were mock or RSV-infected (MOI 1.0). The RSV was left in the media for 24 h until harvest.

### 2.2 RNA isolation and quantitative real time-PCR

Total cellular RNA was extracted with RNeasy kit (Qiagen 75144). cDNA were synthesized with LunaScript RT supermix kit (NEB E3010) and amplified with NEBNext high-fidelity 2x PCR master mix (NEB M0541). For PCR with Taqman primers, Taqman universal PCR master mix (Thermo 4304437) was used. PCR was using an AriaMx real-time PCR machine (Agilent Technologies). Quantification of relative changes in gene expression was calculated using the ΔΔCt method and normalized to internal control peptidylprolyl isomerase A (PPIA). Primer sequences are listed in **Table 1**. Taqman primers are from Thermo: TP63 (Hs00978340_m1), FOXJ1(Hs00230964_m1), SCGB1A1(Hs00171092_m1), MUC5AC(Hs01365616_m1), PPIA(Hs04194521_s1).

**Table 1:** Quantitative XCHP g-PCR and RT-PCR primers.

Gene	Forward	Reverse
**IFITM1-P**	5′-TTGGTCCCTGGCTAATTCAC-3′	5′-TTGTGCATCTTCTGGCTTTG-3′
**BST2-P**	5′-GGGCCACCCCTTTATTAGCA-3′	5′-CGGCCTAGGCACTCAGTAAC-3′
**TLR3-P**	5′-ACGCCTCTCTGAGGTTGTCA-3′	5′-CTGCCCAGTAAGAACGTGAA-3′
**IRF1**	5′-AGCTCAGCTGTGCGAGTGTA-3′	5′-TAGCTGCTGTGGTCATCAGG-3′
**BST2**	5′-CAGAGAAGGCCCAAGGACAA-3′	5′-GTCCGCGATTCTCACGCTTA-3′
**TLR3**	5′-GTTTTCGGGCCAGCTTTCAG-3′	5′-AGAGTTCAAAGGGGGCACTG-3′
**IFITM1**	5′-GGCTTCATAGCATTCGCCTACTC-3′	5′-AGATGTTCAGGCACTTGGCGGT-3′

P, promoter sequence.

Shown are forward and reverse primers in 5’-3’ orientation.

### 2.3 IRF1 CRISPR-Cas9 knockdown

The lentiCRISPRv2 system (Addgene, #52961) was used to prepare IRF1 gene knockout plasmid, with the sgRNA target sequence 5**’**-CACCTCCTCGATATCTGGCA-3**’** targeting exon 4. Together with packaging plasmids psPAX2 (Addgene, #12260) and pCMV-VSV-G (Addgene, #8454), lentiviruses were made in 293T cells with lipofectamine 2000 transfection reagent (Thermo, # 11668030). hSAEC were infected with virus supernatant in the presence of 10ug/ml polybrene and selected 48** h** later with 10 μg/ml of puromycin in culture medium.

### 2.4 Expression plasmids

A lentiviral vector expressing C-terminal FLAG-tagged human IRF1 was generated by cloning human IRF1-3x FLAG coding sequence by PCR into a lentiviral expression vector with CMV promoter to drive target gene expression and harboring Hygromycin-resistant selection marker. Silent mutations were introduced in the IRF1 coding sequence at the IRF1 CRISPR/Cas9 gRNA target sequence by overlap extension PCR. The IRF1 coding sequence at the gRNA target sequence: 5**’**-C*TG CCA GAT ATC GAG GAG GTG*-3**’** (wild-type) vs 5**’**-C*TT CCT GAC ATA GAA GAA GTT*-3**’** (mutant with silent amino acid substitutions).

### 2.5 Western blot

Cells were trypsinized, pelleted and washed twice with cold phosphate-buffered saline (PBS). Cell pellets were then lysed in cold low ionic strength buffer (50 mM NaCl, 1% IGEPAL, 10% Glycerol, 10 mM HEPES, pH7.4) with fresh added proteinase inhibitor cocktail (1:100 vol/vol dilution, Sigma P8340), 1 mM DTT and 1 mM PMSF (Sigma P7626). After determination of protein concentration, samples were denatured in SDS sample buffer, heated at 95°C for 5 min, and fractionated using Criterion TGX 4-15% PAGE gels. Proteins were transferred to PVDF membranes and probed with anti-IRF1 (Proteintech 11335-1-AP, 1:500), anti-FLAG M2 (Sigma F1804, 1:1000) or anti-actin (937215, R&D, 1:5000) antibodies (Ab). Anti-TBP Ab (Biolegend 668306, 1:1000) was used as a loading control. Blots were imaged and bands quantified.

### 2.5 Immunofluorescence microscopy and Electron microscopy

hSAECs were plated on coverslips, and infected or treated as indicated. Afterwards, cells were fixed with 4% paraformaldehyde, permeabilized with 0.1% Triton X-100, blocked with 5% goat serum and incubated with primary Ab overnight (TP63: Abcam ab124762, 1:200; KRT5: Abcam ab52635, 1:100; IRF1: Proteintech, 11335-1-AP, 1:200). On the second day, coverslips were washed with 0.2% Tween-20 and incubated with Alexa fluor goat secondary Ab. After one hour, cells were washed and mounted using ProLong Diamond Antifade Mountant with 4′,6-diamidino-2-phenylindole (DAPI, Thermo Fisher P36931). The cells were visualized in an ECHO Revolve fluorescence microscope.

For electron microscopy, cells were fixed with Karnovsky’s fixative (Electron Microscope Sciences, 15720). Fixed cells were dehydrated in a graded ethanol series followed by dehydration in propylene oxide, then embedded in Epon epoxy resin. Ultra-thin sections were cut with a Leica EM UC6 Ultramicrotome and collected on pioloform-coated 1 hole slot grids (Ted Pella Inc, cat # 19244). Sections were contrasted with Reynolds lead citrate and 8% uranyl acetate in 50% EtOH. Ultrathin sections were visualized with a Philips CM120 electron microscope and images were captured with an AMT BioSprint side mounted digital camera using AMT Capture Engine software.

### 2.6 Proximity ligation assay

PLA was performed with Duolink *in situ* red starter mouse/rabbit kit (Sigma DUO92101). Cells were treated as described in text, fixed with 4% paraformaldehyde, permeabilized with 0.1% Triton X-100, and incubated overnight with indicated pairwise combinations of mouse IRF1 Ab (R&D MAB4830-SO, 1:250), rabbit BRD4 Ab (Cell Signaling 13440, 1:500), rabbit phosphorylated Ser2 CTD RNA Pol II Ab (Abcam ab5095, 1:700), or mouse anti-FLAG M2 Ab (Millipore F1804, 1:300). PLA of BRD4 and p-pol II was done in hSAECs transfected with FLAG-BRD4 lentivirus, containing the coding sequences from FLAG-BRD4 plasmid (Addgene 90331).

Cells were further processed according to the manufacturer’s instructions. The nuclei were counterstained with DAPI, and the PLA signals were visualized with an ECHO fluorescent microscope at x 20 magnification.

### 2.7 Two-step chromatin IP-Quantitative genomic PCR

Three 100 mm dishes of cells each treatment group were first cross-linked with disuccinimidyl glutarate (DSG; 2 mM, 45 min at 22°C, Thermo 20593), then cross-linked with methanol-free formadehyde (1% vol/vol in PBS, 10 min). Cells were extracted in SDS lysis buffer and sonicated. Equal amounts of sheared chromatin were immunoprecipitated (IP) overnight at 4°C with 3 μg of Ab. The following antibodies were used: IRF1 (Proteintech 11335-1-AP), BRD4 (Cell Signaling 13440), p-Ser2 Pol II (Abcam ab5095); IgG was a nonspecific binding control (LS Bio LS-C149375). IPs were collected with 20 μl of Dynabeads protein G (Thermo 10004D), washed, eluted and de-crosslinked. The precipitated DNA was phenol/chloroform-extracted, precipitated and air-dried. Gene enrichment was determined by Q-gPCR using promoter-specific PCR primers. Primer sequences are listed in **Table 1**. The fold change of precipitated DNA was determined by normalizing to input DNA and calculating the fold change relative to the amount in unstimulated cells (37, 38).

### 2.8 Statistical analyses

RNA-seq was quantitated from Illumina High Seq fastq files. The raw fastq files were subjected to QC statistics and sequencing adapters were trimmed using Trim Galore software (39). The trimmed paired-end reads were aligned against human genome hg38 using Salmon (40). Mapped paired-end reads for both genes and transcripts (isoforms) were counted in each sample and variance stabilized using DESeq2 (33). Statistical analyses were performed with Graph Pad Prism 9 (GraphPad Software, San Diego, CA). Results are expressed as mean ± SD. Normality and equal variance tests were performed to determine appropriate application of parametric statistical analyses. For multiple group experiments, one-way ANOVA was used with post-hoc Tukey T-tests for group-wise comparison between treatments. P values < 0.05 were considered to be statistically significant.

## 3 Results

### 3.1 hSAECs are model multipotent airway stem cells

We investigated the presence of the intrinsic IIR in human small airway epithelial cells (hSAECs), a well characterized small airway-derived basal cell (41, 42). Partially permissive for RSV replication, hSAECs undergo TGFβ- and RSV replication-induced changes in cellular plasticity (13), a cell-state change characterized by simultaneous expression of epithelial mesenchymal transition (EMT) and mesenchymal-epithelial transition (MET) markers, activating metabolic reprogramming and extracellular matrix remodeling characteristic of *in vivo* RSV-infections (34, 42).

**Figure 1:**
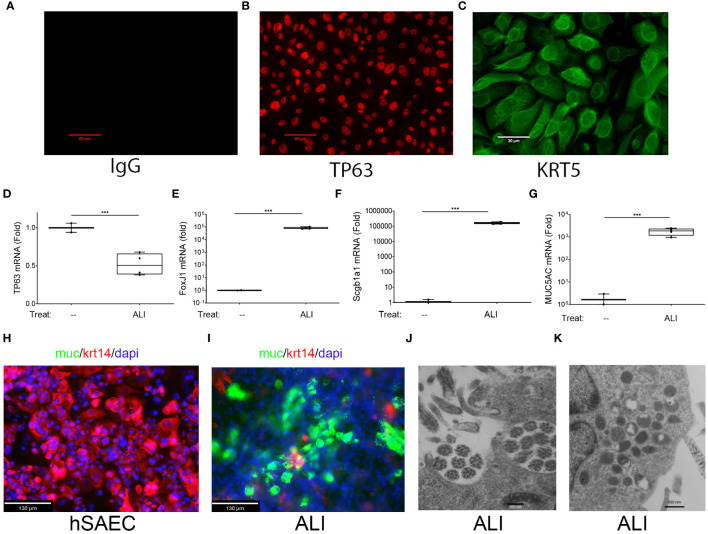
Basal cell behavior of hSAECs. **(A–C)** hSAECs were plated on coverslips, fixed and stained with IgG panel **(A)**, anti-TP63 panel **(B)** or anti-KRT5 Abs panel **(C)**. Shown are immunofluorescence microscopy (IFM) images. Scale bars for panels **(A, B)** are 60 μm; Scale for panel **(C)** is 30 μm. **(D–G)** hSAECs were cultured ± air liquid interface (ALI) and expression of basal and differentiated epithelial cell markers were analyzed by Q-RT-PCR. **(D)** *TP63* mRNA; **(E)** *FOXJ1* mRNA; **(F)** *Scgb1a1* mRNA; **(G)** *MUC5AC* mRNA. Shown are fold change mRNA normalized to *PPIA*. Each symbol represents an independent replicate. ***, P<0.001. **(H, I)** IFM. Submerged cultures of hSAECs or those from ALI culture were stained with anti-Mucin (Muc, green) and KRT14 (red) Abs. DAPI (blue) is used as counterstain. Scale bar is 130 μm. **(J, K)** Transmission electron microscopy of ALI cultures. **(J)** Microtubule cluster characteristic of differentiated cilia. Scale bar is 200 nm. **(K)** Electron dense secretory granules, characteristic of club cells. Scale bar is 500 nm.

Because of their ability to enter cellular plasticity programs, we examined whether hSAECs exhibited airway stem-cell features. First, we stained hSAECs for tumor protein 63 (TP63), a characteristic basal cell marker and nuclear factor essential for endodermal differentiation (43), by immunofluorescence microscopy (IFM). Relative to negative control staining with IgG, we observed uniform nuclear TP63 staining in >95% of cells (**Figures 1A, B**). Similarly, hSAECs expressed high levels of keratin 5 (KRT5), a keratin isoform expressed by basal airway epithelial cells, in a perinuclear and cytoplasmic microfilamentous pattern (**Figure 1C**). The presence of these markers are significant because TP63+/KRT5+ expressing progenitors from the bronchiolar epithelium repopulate the distal airway and type I and II alveolar cells after viral injury (23, 24) and are therefore characteristics of airway basal cells.

To confirm that hSAECs had multipotent tissue stem-cell activity, hSAECs were induced to differentiate in air liquid interface (ALI). After 21d in culture, hSAECs reduced *TP63* mRNA expression by 50% (**Figure 1D**) and acquired markers of ciliated (FoxJ1), club (Scgb1a1) and mucin-producing (MUC5a) differentiated airway cells, with a 5-fold log increase in *FoxJ1*, 5-fold log increase in *Scgb1a1* and 3-fold log increase in *MUC5AC* transcripts (all P<0.001; **Figures 1E–G**).

By IFM, in submerged hSAEC cultures, cells were MUC negative (MUC^-^) and KRT14 positive (KTRH^+^; **Figure 1H**). KRT14 is another keratin isoform expressed by basal cells of the airway. By contrast, after 21 d in ALI, hSAECs exhibited strongly positive MUC5AC staining in a heterogenous pattern with reduced KRT14 expression (**Figure 1I**). To confirm the presence of ciliated structures and club-cell like secretory granules, electron microscopy was performed. Here paired microtubules forming a classical ciliated structure are seen (**Figure 1J**). In other cells, cytoplasmic electron-dense secretory vesicles typical of Scgb1a1 positive club cells were also seen (**Figure 1K**). We conclude that hSAECs maintain basal cell like characteristics, that functionally differentiate into differentiated ciliated, club and mucous expressing epithelial cells.

### 3.2 hSAECs exhibit the “intrinsic” IIR program of tissue stem cells

**Figure 2:**
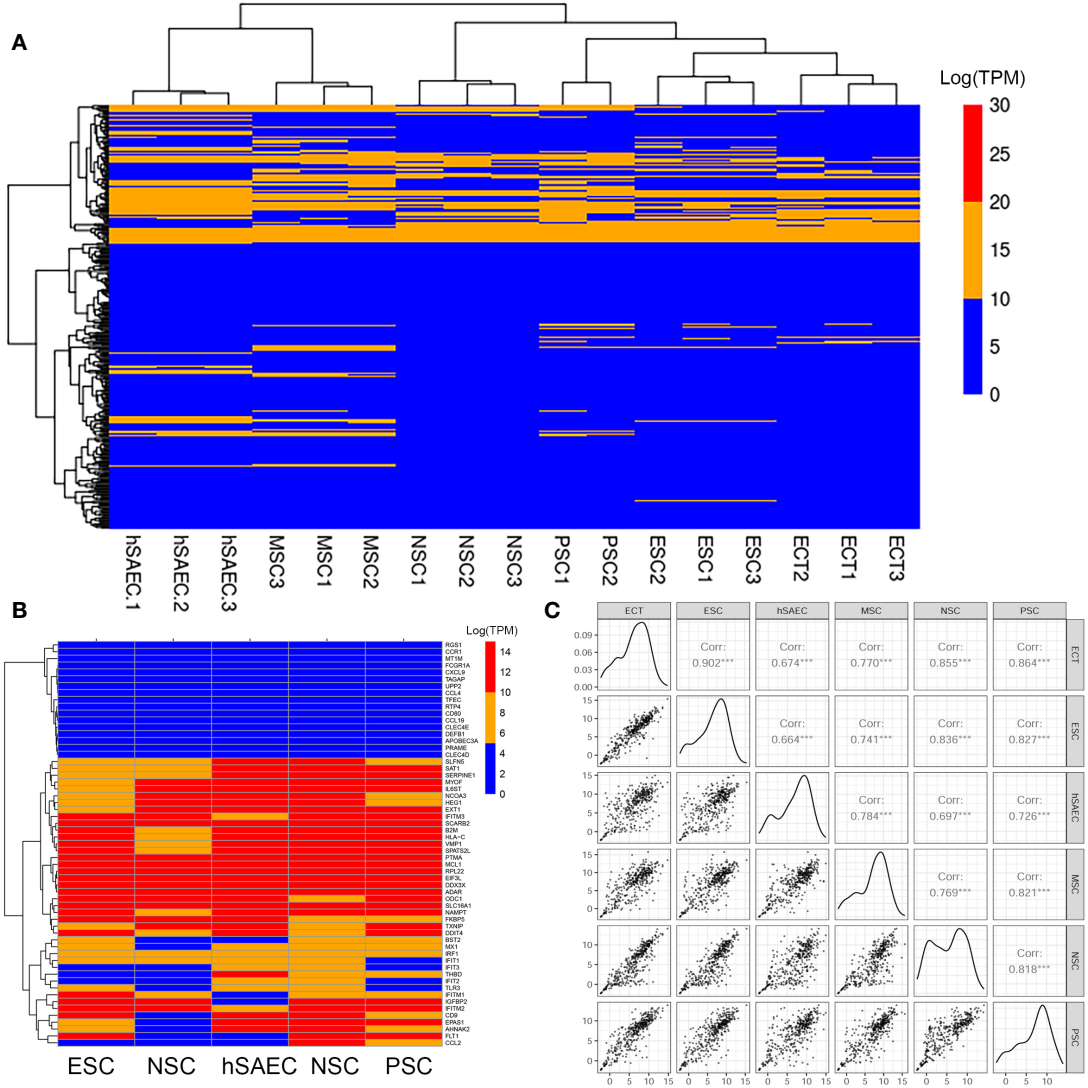
hSAECs express a conserved innate response. **(A)** Expression patterns of IFN stimulated genes (ISG) across pluripotent and tissue stem cells. Shown is hierarchical clustering (HC) of mRNA expression (in log-stabilized TPM) of 298 ISGs for hSAECs *vs* multipotent ESCs and multipotent tissue stem cells. MSC, Mesenchymal stem cells; NSC, neuronal stem cells; PSC, pancreatic stem cells; ESCs, ectodermal stem cells. Legend of expression values is shown at right. **(B)** HC of type I and III ISGs. Each row represents a gene, labeled at right. **(C)** Pairwise scatter plot of all ISGs for hSAECs *vs* multipotent ESCs and multipotent tissue stem cells. Note the high degree of concordance.

The intrinsic IIR pathway consists of a ~100 member subset (of the ~390 known ISGs) that are constitutively expressed. The intrinsic IIR includes *BST2*, *IFIT and TLR3* encode broad-anti-viral activities shown to be functionally important in conferring anti-viral defense, including limiting RSV replication (25). To determine whether hSAECs expressed a similar intrinsic innate program, we compared RNA-Seq profiles of hSAECs to those of pluripotent embryonic stem cells (ESC) and tissue-derived stem cells committed to mesenchymal (MSC), neuronal (NSC) and pancreatic (PSC) lineages (25, 42). In this comparison, RNA-seq was quantitated from 3 independent replicates by transcripts per million (TPM), normalized to read depth and log-transformed using variance stabilization in DESeq2 (44). The ISG expression patterns were then subjected to hierarchical clustering. We observed that the expression patterns were quite similar with hSAECs most closely grouping with MSC cell types (**Figure 2A**). A highly expressed set of ISGs was consistent across all cell types; these genes included genes of the anti-viral *IFIT* cluster (*IFIT1M-1,-2,-3)* as well as the transcription factors *IRFs 1* and *-9* (**Figure 2A**). This global expression showed that the intrinsic IIR pathway was remarkably similar across cell types examined.

To better visualize the expression of *IFIT*-enriched core of genes, we extracted a focused group of anti-RSV ISGs and found that these expression patterns were remarkably consistent across the cell types tested (**Figure 2B**). To provide more quantitative, global information on the relationship between intrinsic IIR expression levels, pairwise correlations of all 390 known ISGs were conducted across the same pluripotent and multipotent stem cell data. We found that the correlation across these tissue stem cells was remarkably linear across all pairwise comparisons (**Figure 2B**). Linear regression modeling produced with a 95% linear regression line approaching a slope of 1, that was highly statistically significant (P<10^-16^; **Figure 2C**). Collectively, we conclude that hSAECs exhibit morphological and functional characteristics of multi-potent airway stem cells and express the highly conserved intrinsic IIR program.

### 3.3 IRF1 maintains constitutive expression of intrinsic innate response genes

**Figure 3:**
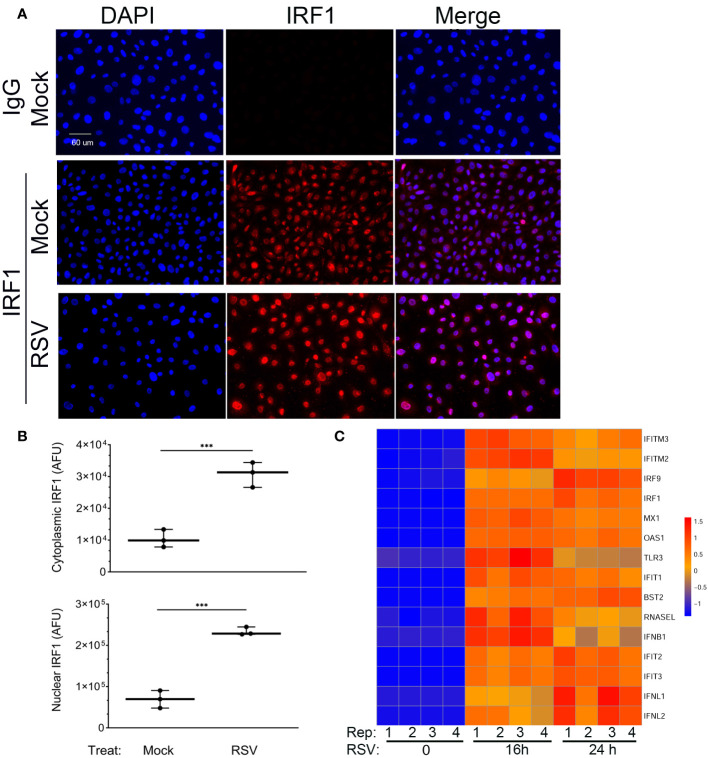
Constitutive and RSV inducible expression of IRF1. **(A)** IFM was performed in mock or RSV-infected hSAECs with IgG or anti-IRF1 (red) and nuclei stained with DAPI (blue). Shown are DAPI and IRF1 images in single channel images or as a merged image. Scale bar, 60 μm. **(B)** Quantitation of IRF1 nuclear and cytoplasmic staining. Images were segmented and nuclear intensity analyzed by FIJI. Shown is cytoplasmic (top) and nuclear (bottom) IRF1 fluorescence in arbitrary fluorescence units (AFUs). Each point is the aggregate staining of an independent high-power field consisting of >50 cells. **(C)** Intrinsic IIR genes are highly inducible in hSAECs. A time course of RNA seq profiles was analyzed in cells infected by RSV for 0 h, 16 h and 24 h (n=4 replicates). RNA seq data was scaled and visualized by hierarchical clustering. Note high level of mRNA inducibility in response to RSV infection. ***, P<0.001.

We noted that many of the intrinsic IIR genes are, at least in part, IRF1-dependent (30). Since these genes have constitutive expression in mock-infected hSAECs, we examined the expression of nuclear IRF1 using dual color staining for IRF1 and nuclei (DAPI) in IFM. We found that IRF1 was expressed in both the cytoplasm and nucleus of mock-infected hSAECs (**Figure 3A**). Upon RSV infection, the nuclear and cytoplasmic abundance of IRF1 was increased. In mock-infected hSAECs, IRF1 nuclear staining intensity was 69,323 ± 21,290 arbitrary fluorescence units (AFUs) and increased 3.4-fold to 233,307 ± 9885 AFUs after RSV infection (**Figure 3B**; P=0.0003, two-tailed t test). A similar 3-fold induction was also seen in cytoplasmic IRF1 staining in response to RSV infection (P<0.001; **Figure 3B**).

To determine whether the basal level of IRF1 expression produced saturating expression of the intrinsic innate response, we extracted gene expression profiles of ISGs over a time course of RSV infection from an earlier RNA-Seq data set. In this analysis, we performed Z-score normalization of the RNA-Seq expression profiles in order to compare of each gene expression profile to its own uninfected baseline. This hierarchical clustering analysis showed that all the intrinsic response genes are induced by 16- and 24 h of RSV infection (**Figure 3C**). Collectively these data indicate that the IIR genes are still highly inducible despite constitutive IRF1 expression.

### 3.4 Ambient IRF1 is rate-limiting for expression of a core of intrinsic IIR genes

To better understand the role of IRF1 in direct activation of intrinsic IIR genes, we first sought to identify those directly regulated by IRF1. This is important because our previous work has shown that IRF1 amplifies the expression of other IRF-family transcription factors through positive autoregulatory loops (45), making identification of genes directly regulated by IRF1 challenging. To more rigorously identify direct targets of IRF1, we conducted experiments to identify genes whose expression is IRF1 dependent (by silencing and overexpression) and genes that directly bound IRF1 to their proximal promoters in chromatin immunoprecipitation.

**Figure 4:**
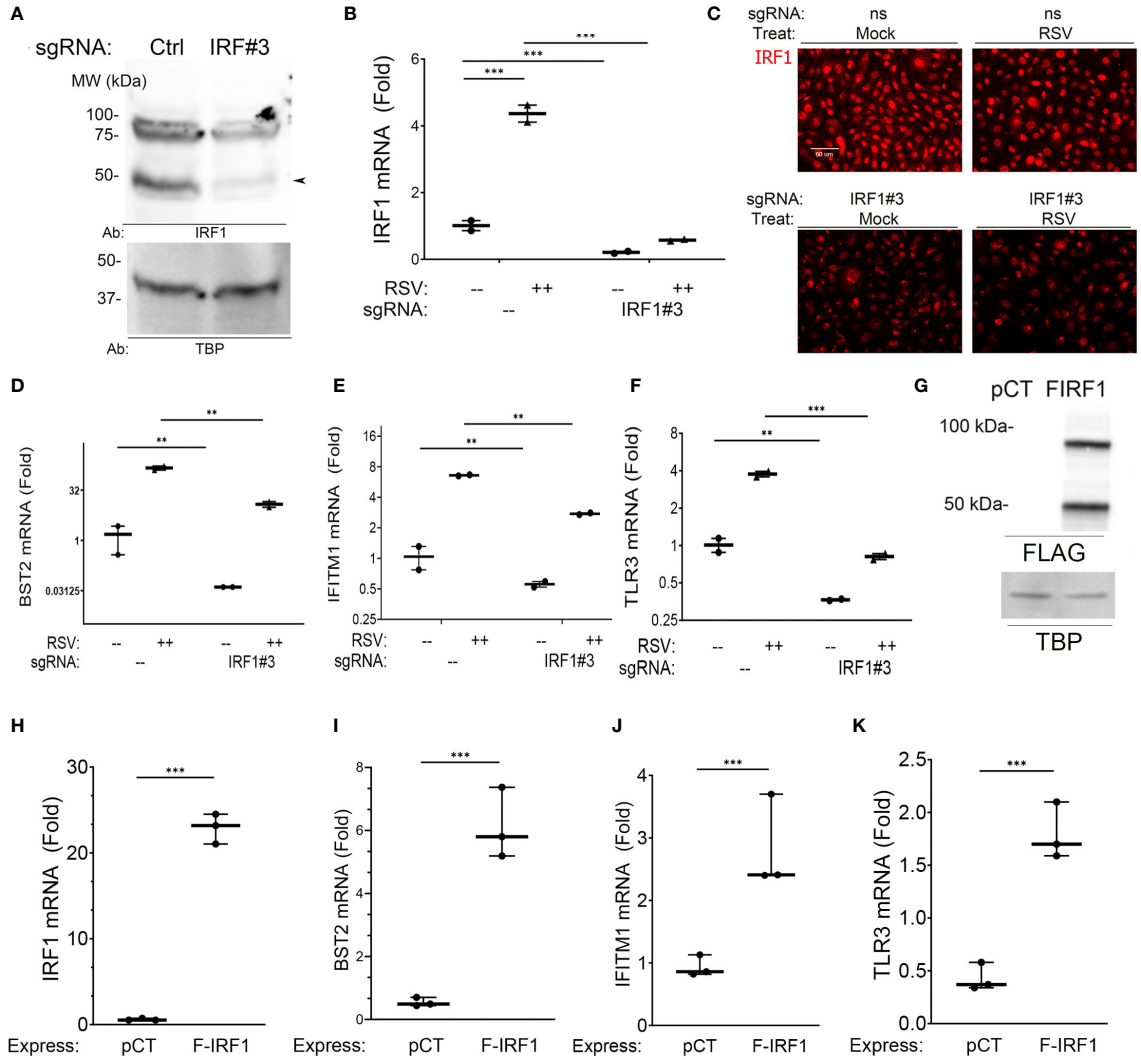
Intrinsic innate response genes are directly regulated by IRF1. **(A)** IRF1 KD. Western blot of nuclear extracts from nontargeting control (–) or IRF1 (IRF#3) -sgRNA transfectants. Left, MW markers in kDa. Arrowhead, 50 kDa IRF1 isoform. **(B–F)** Effect of IRF1 Crispr/Cas9-mediated knock down (KD). mRNA expression was quantified by Q-RT-PCR. Inducible expression of intrinsic innate response genes was analyzed by Q-RT-PCR. **(B)** *IRF1* mRNA; **(C)** IRF1 in immunofluorescence microscopy; **(D)** *BST2* mRNA; **(E)** *IFITM1* mRNA; and **(F)** *TLR3* mRNA. Shown is fold change mRNA normalized to internal control *PPIA* plotted as log2-transformed data. **, P<0.01; ***, P<0.001. Note the reduction in basal mRNA expression by IRF1 KD. **(G)** Western blot of FLAG-IRF1 (F-IRF1) lentiviral expression in IRF1 KD cells. pCT, empty lentiviral construct. FIRF1, FLAG-IRF1 expression. TBP, internal loading control. **(H–K)** Effect of FLAG-IRF1 expression on intrinsic IIR genes. Q-RT-PCR fold change mRNA expression is shown.

We first conducted IRF1 knockdown (KD) experiments using CRISPR/Cas9. Three small guide RNA (sgRNAs) were screened for efficiency of IRF1 KD; sgRNA#3 produced the most significant inhibition and was used. Control (nontargeting) and IRF1-targeting sgRNAs were transfected into hSAECs and then mock- or RSV infected (MOI=1, 24 h). In the nuclei of control sgRNA-transfectants, several isoforms of IRF1 protein were detected, at 50- and a ~100 kDa doublet (**Figure 4A**). These two larger isoforms are IRF1 homodimers or alternatively spliced IRF isoforms (46). All proteoforms were substantially reduced by the IRF1-targeting sgRNA transfection (**Figure 4A**).

Similarly, constitutive *IRF1* mRNA was reduced by >80% in IRF1-targeting sgRNA-transfected cells (**Figure 4B**). And RSV infection induced a 4.5-fold induction of *IRF1* mRNA expression in nontargeting sgRNA transfected cells. This induction was reduced to less than that of mock-infected controls after IRF1 sgRNA transfection (**Figure 4B**). Similarly the constitutive and RSV-induced nuclear IRF1 signals were substantially reduced by IFM assay (**Figure 4C**). This system enabled us to determine the role of IRF1 in constitutive and inducible intrinsic innate response.

We found that constitutive *BST2* mRNA levels were reduced by 37-fold in mock-infected IRF1 KD cells, where the 1.5 ± 1.6 fold *BST2* expression in control transfectants was reduced to 0.04 ± 0.001 fold in IRF1 KDs (**Figure 4D**). And in RSV-infected cells, the 149 ± 29-fold induction of *BST2* mRNA was reduced to 12.7± 3.3 -fold in IRF1 KD cells (P<0.001). Similarly, the constitutive expression levels of *IFITM1* were reduced 1.7 fold vs IRF1 KD cells (**Figure 4E**), and constitutive levels of *TLR3* were reduced 3-fold in IRF1 KD cells (**Figure 4F**). Significant reductions in RSV-induced *IFITM1* and *TLR3* mRNA expression were also observed by IRF1 KD (**Figures 4D–G**).

To independently test whether IRF1 expression was sufficient for expression of this intrinsic innate genes, FLAG-IRF1 was expressed in IRF1-deficient hSAECs. Here, empty lentivirus (pCT) or FLAG-epitope labeled IRF1 expressing lentivirus were used to transduce IRF1 KD cells, where both 50- and 100 kDa isoforms of IRF1 were observed (**Figure 4G**). Transfection of the FLAG-IRF1 construct produced a 22 ± 1.8-fold induction of mRNA, exceeding the 4-fold induction of endogenous cells (cf., **Figures 4H, B**). Under these conditions, we observed that FLAG-IRF1 expression increased *BST2* mRNA only by 6.1 ± 1.1-fold (P<0.001, **Figure 4I**). We also noted that *IFITM1* mRNA increased by 2.8 ± 0.8-fold (P<0.05, **Figure 4J**) and *TLR3* mRNA was enhanced by 1.8 ± 0.3-fold after FLAG-IRF1 expression (P<0.05, **Figure 4K**). Collectively, these data indicate that *BST2, IFITM1* and *TLR3* core intrinsic IIRs are maintained in a low level of expression by ambient IRF1, and although poised for high inducibility, their maximal expression could not be reproduced by IRF1 alone.

Finally, to demonstrate that these genes are direct IRF1 targets, chromatin immunoprecipitation experiments (XChIP) were performed. Here, cross-linked chromatin from mock or RSV-infected wild-type hSAECs were immunoprecipitated with non-specific IgG or IRF1 Abs, and the abundance of the *BST2, IFITM1* and *TLR3* proximal promoters were quantitated by quantitative genomic PCR (Q-gPCR).

**Figure 5:**
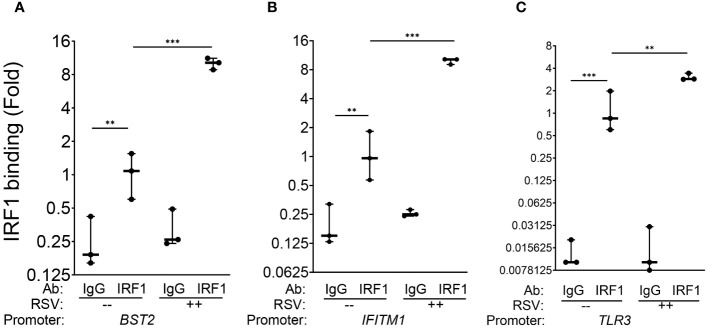
IRF1 constitutively and inducibly engages with intrinsic innate response genes. XChIP assays were performed on mock or RSV-infected hSAECs (MOI 1, 24 h). Cross-linked chromatin was immunoprecipitated with IgG or anti-IRF1 Abs and proximal promoter enrichment determined by quantitative genomic PCR. Shown is fold enrichment of each biological replicate normalized to IRF1 IPs of mock infected cells. Data are plotted by log2 transformation to better visualize the dynamic changes in IRF1 abundance. **(A)** *BST2* promoter; **(B)** *IFITM1* promoter; **(C)** *TLR3* promoter. **, P<0.01; ***, P< 0.001.

We first estimated the binding of IRF1 to these genes in mock-infected cells, where gene enrichment from IRF1 immunoprecipitates was estimated relative to background IgG binding. In uninfected hSAECs, we observed a 4.2-fold enrichment of IRF1 binding to the *BST2* promoter over IgG background by Q-gPCR (**Figure 5A**). Similarly, we found a 6-fold enrichment in constitutive IRF1 binding on the *IFITM1* promoter over IgG background (**Figure 5B**) and an 8.7-fold increase of IRF1 was observed on the *TLR3* promoter (**Figure 5C**).

We then estimated RSV-induced changes in IRF1 binding by comparing Q-gPCR signals of IRF1 immunoprecipitates from RSV *vs* mock-infected cells. We noted that RSV infection further enhanced IRF1 binding to the *BST2* promoter by 10.1 ± 1.2-fold (P<0.001, **Figure 5A**). Similarly, RSV induced IRF1 binding to the *IFITM1* promoter by 9.8 ± 0.7 fold (P<0.001, **Figure 5B**); and IRF1 binding to the *TLR3* promoter by 3.1 ± 0.3-fold relative to mock infected cells (P<0.01, **Figure 5C**). Collectively, we conclude that IRF1 is constitutively engaged with the core intrinsic IIR genes and is further induced by RSV in a rate-limiting fashion.

### 3.5 IRF1 interacts with activated RNA Pol II

In differentiated epithelial cells, viral inducible ISG expression is controlled by regulated transcriptional elongation (29, 31, 32, 45, 47–53). In uninfected cells, ISGs are transcriptionally silenced, but maintained in an open chromatin configuration engaged by paused RNA Pol II. In response to infection, viral activated transcription factors recruit the PTEF-b complex containing cyclin dependent kinases and BRD4 to phosphorylate the carboxyl terminal domain (CTD) of RNA Pol II on Ser residue 2. Ser2 phosphorylation licenses the stalled polymerase to enter rapid processive mode, rapidly expressing fully spliced anti-viral transcripts (31, 49). Because the intrinsic immune genes are constitutively transcribed in hSAECs in an IRF1-dependent manner, we hypothesized that IRF1 was engaged with phospho-Ser 2 RNA Pol II (pSer2 Pol II) to control their metastable state.

**Figure 6:**
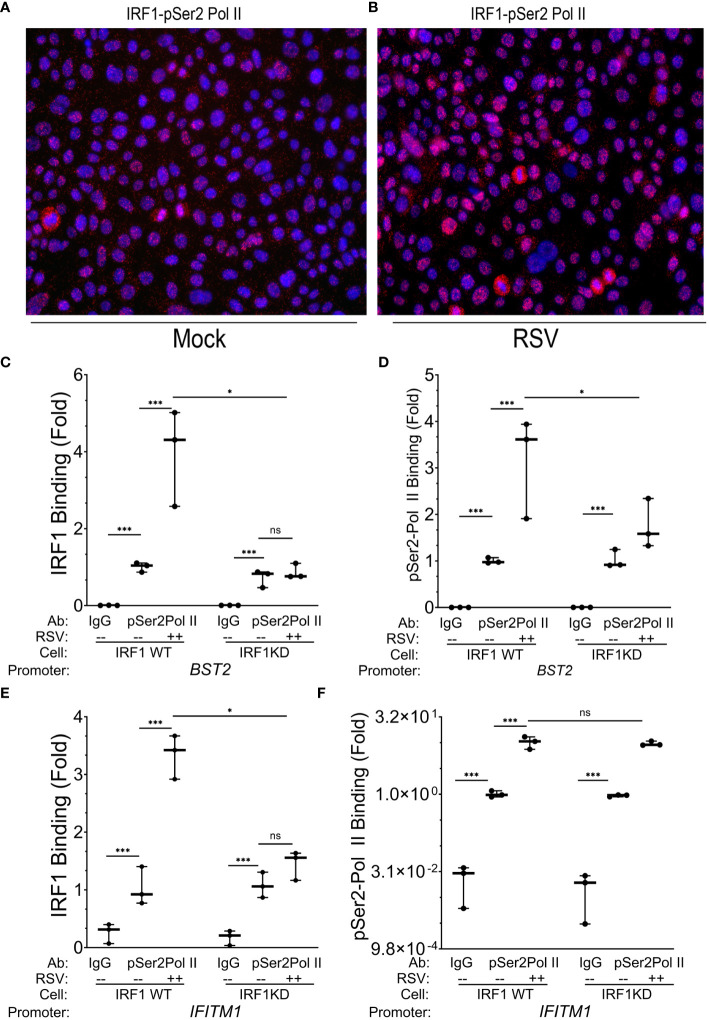
IRF1 directly engages with pSer2 Pol II. **(A, B)** Proximity ligation assays were performed with mouse anti-IRF1 and rabbit anti-pSer2 Pol II Abs on mock or RSV infected hSAECs (MOI 1, 24 h). After secondary antibody reaction and PCR amplification, regions of interaction are indicated by red dots. Nuclei are counterstained with DAPI (blue). Scale marker is 30 μm. **(C–F)** Wild type (WT) or IRF1 KD hSAECs were mock or RSV infected (MOI 1, 24 h). XChIP was performed to measure IRF1 and pSer2 Pol II binding to **(C, D)** *BST2* and **(E, F)** *IFITM1* proximal promoters. Shown are fold enrichment by Q-gPCR. *IFITM1* data are expressed in scientific notation to illustrate the dynamic range of pSer2 Pol II binding. **, P<0.01; ***, P< 0.001; n.s., not significant.

We first determined whether IRF1 and pSer2 Pol II interacted in uninfected cells using proximity ligation assays (PLAs; **Figures 6A, B**). PLAs detect atomic-distance interactions *in situ* detected by the enzymatic ligation of separately directed antibody-conjugated oligonucleotides that are amplified by PCR, appearing as red foci in immunofluorescence (54, 55). Strikingly, we found that IRF1 is complexed with pSer2 Pol II in >90% of uninfected hSAECs (~450 cells counted in 3 independent low-power images; **Figure 6A**; note that no signal is produced in cells stained with IgG; c.f. **Figure 3A**). We also observed the number of nuclear IRF1-pSer2 Pol II foci were enhanced 3.8-fold by the effect of RSV infection, from 6.3 ± 4.1 to 24.0 ± 4.7 foci/nucleus (**Figure 6B**, P=0.0013, two-tailed t test).

To determine whether pSer2 Pol II directly binds intrinsic response genes, and if pSer2 Pol II binding was dependent on IRF1 binding, we performed XChIP on wild type (WT) and IRF1 KD cells. We confirmed that IRF1 was constitutively bound to the *BST2* gene promoter in mock-infected cells, and was induced ~4-fold in response to RSV infection (**Figure 6C**). Although the level of basal IRF1 was resistant to IRF1 KD, RSV inducible recruitment was substantially inhibited in IRF1 KD cells to levels seen in mock-infected cells (**Figure 6C**).

Examining the presence and regulation of pSer2 Pol II binding to *BST2*, we observed a 50-fold enrichment of constitutive pSer2 Pol II signals over that of control IgG in mock-infected WT cells (0.002 ± 0.0002 in IgG precipitates vs 1.02 ± 0.01 in pSer2 Pol II precipitates; **Figure 6D**). In response to RSV infection, pSer2 Pol II binding increased 3.1 ± 1.1- fold in WT cells (**Figure 6D**). Importantly, the level of RSV induced pSer2 Pol II was substantially reduced in IRF1 KD cells to 1.8 ± 0.5-fold, indicating that recruitment of pSer2 Pol II binding to *BST2* was dependent on IRF1 (**Figure 6D**).

Similarly, we observed 3.3 ± 0.4-fold induction of IRF1 binding to the *IFITM1* promoter in WT cells that was reduced to 1.5 ± 0.4-fold after IRF1 KD (P<0.05, **Figure 6E**). Next we examined pSer2 Pol II binding to the *IFITM1* promoter and found that significant constitutive levels of pSer2 Pol II binding, with 1.0 ± 0.14 fold pSer2 Pol II immunoprecipitates vs 0.024 ± 0.02 fold in IgG precipitates (**Figure 6F**). pSer2 Pol II binding was highly inducible by RSV, increasing by 10.1± 2.8-fold in WT cells. Interestingly, in contrast to that seen on *BST2*, this high level of RSV-induced pSer2 Pol II binding was not significantly affected by IRF1 KD (**Figure 6F**). These data suggested that there were both IRF1 dependent and independent modes of pSer2 Pol II recruitment to the intrinsic IIR genes.

### 3.6 BRD4 chromatin remodeling complex is inducibly recruited to intrinsic response genes

The findings that IRF1 expression alone is insufficient to fully activate the intrinsic IIR to levels seen by RSV, and the pSer2 Pol II recruitment is IRF1-independent suggested to us that additional activation mechanisms controlling regulated transcriptional elongation are playing a role. Our earlier work implicated the atypical acetyltransferase BRD4 as important mediator for recruitment of RNA polymerase -directed kinases to target genes, promoting transcriptional elongation (29, 45, 56–58). These studies elucidated the interaction of BRD4 with innate-responsive NFκB/RelA and activating protein-1 transcription factor (59). However, the effects of BRD4 interaction with IRF1 are not fully understood.

**Figure 7:**
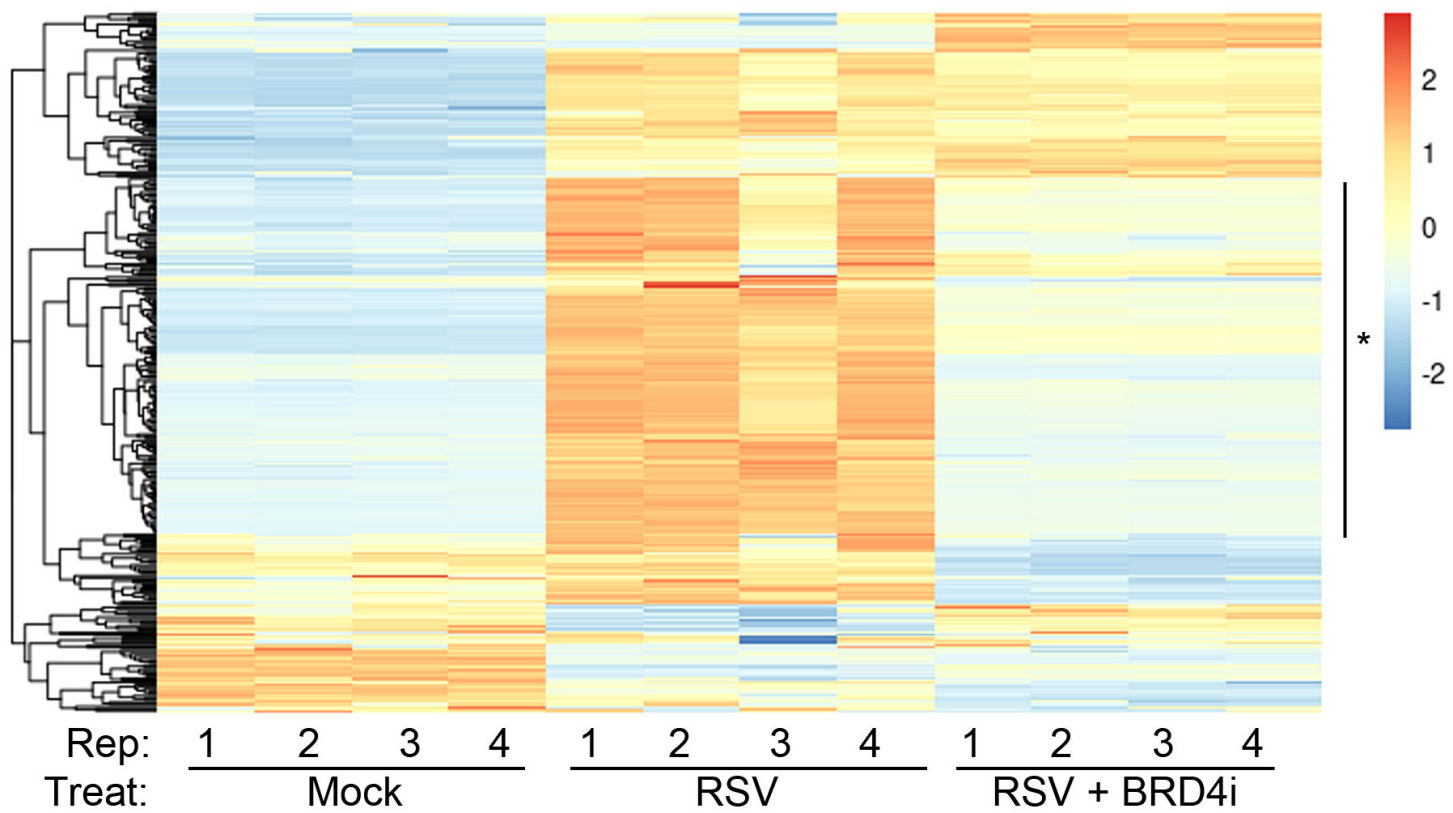
BRD4 is a master controller of the induced intrinsic innate response. Hierarchical clustering of RNA-Seq from WT hSAECs ± BRD4i treatment was performed. Four biological replicates of RNA-Seq from three hSAEC treatment conditions were analyzed: 1. mock-treated hSAECs, 2. RSV infected hSAECs or 3. RSV infected hSAECs treated with BRD4i (ZL0454, 10 μM). Gene expression patterns for the intrinsic innate response genes were extracted, Z-score scaled and subjected to hierarchical clustering. The major cluster (*) is highly induced by RSV infection and BRD4 dependent.

We therefore explored the functional interaction between BRD4 and IRF1. Highly selective small molecule inhibitors of BRD4 (BRD4i) have been developed that enable the study of the functional role of BRD4 bromodomain interactions (53, 60–62). Examining the effect of BRD4i on ~390 known ISGs, we found that a large cluster of >150 intrinsic IIR genes were highly RSV inducible and sensitive to the effect of BRD4i (**Figure 7**).

**Figure 8:**
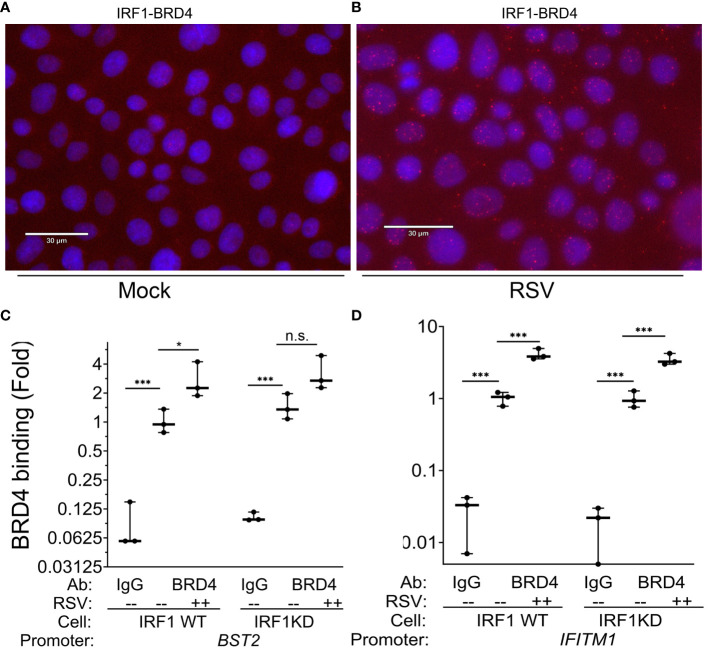
RSV induces IRF1·BRD4 complex formation. **(A, B)** Proximity ligation assays were performed with mouse anti-IRF1 and rabbit anti-BRD4 Abs on mock or RSV infected hSAECs (MOI 1, 24 h). After secondary antibody reaction and PCR amplification, regions of interaction are indicated by red dots. Nuclei are counterstained with DAPI (blue). Scale marker is 30 μm. **(C, D)** IRF1 Wild type (WT) or IRF1 KD hSAECs were mock- or RSV infected (MOI 1, 24 h). XChIP was performed to measure BRD4 binding to **(C)** *BST2* and **(D)** *IFITM1* proximal promoters. Shown are fold enrichment by Q-gPCR. *, P<0.05, ***, P< 0.001.

We next explored the interaction between IRF1 and BRD4 in mock and RSV-infected cell states. Of particular interest here, in mock-infected WT cells, we observed that an IRF1·BRD4 complex was detected in less than 1% of uninfected cells by PLA (0.03 ± 0.3 **Figure 8A**). By contrast, RSV infection increased both the number of IRF1·BRD4 foci/cell and percentage of cells with

IRF1·BRD4 complexes (19.9 ± 13% of cells in 3 high power fields, n=50 cells/field; P=0.02, t-test; **Figure 8B**). These data suggested BRD4 may play a role in RSV-inducible RNA Pol II activation on IRF1 dependent IIR genes.

To determine whether BRD4 is recruited to intrinsic IIR genes and whether the interaction with IRF1 influenced its genomic targeting, XChIP assays were performed in WT and IRF1 KD cells. In mock infected WT cells, BRD4 binding was detected on the *BST2* promoter (0.01 ± 0.1 in IgG *vs* 1.2 ± 0.3-fold in BRD4 IPs, P<0.05, post-hoc), a value which increased to 2.8 ± 1.2-fold after RSV infection (P=0.012, post-hoc, **Figure 8C**). In mock-infected IRF1 KD cells, a similar level of BRD4 binding was seen as that in WT controls, and in response to RSV infection was a trend for increase of BRD4 binding, but this difference was not significant (3.3 ± 1.4-fold, P<0.05, post-hoc; **Figure 8C**).

A distinct pattern of inducible BRD4 interaction was observed on *IFITM1*. Like *BST2*, significant signal of BRD4 binding to the *IFITM1* promoter could be detected in mock-infected cells over that

of IgG controls (P<0.001; **Figure 8D**). In response to RSV, a 4.1 ± 0.7-fold increase in BRD4 binding was produced in WT cells and a 3.5 ± 0.7-fold increase in IRF1 KD cells (P<0.001 vs mock infected KD cells; **Figure 8D**). The increased induction of BRD4 to *IFITM1* was indistinguishable between WT and IRF1 KD cells.

From these data, we conclude that: 1. BRD4 is engaged on intrinsic IIR genes; 2. RSV induces IRF1 to bind the BRD4 CRC; 3. RSV induces BRD4 loading on the IIRs; and 4. BRD4 targeting to the intrinsic IIR gene is largely independent of IRF1.

### 3.7 IRF1 and BRD4 independently cooperate in the intrinsic innate response

We next explored the nature of interaction between IRF1 and BRD4 in the intrinsic innate response. BRD4 contains two highly conserved N-terminal bromodomains (BDs), BD1 and BD2, which mediate RSV-inducible interactions with ~100 proteins including transcription factors, chromatin regulators, spliceosome components, mediator proteins and polymerases (63–65); note in this analysis, IRF1 was not observed in the BRD4 CRC). We hypothesized that BRD4 BD serves to recruit coactivator complexes that function independently of its interaction with the PTEF-b complexes; complexes that interact with the COOH terminal BET domain (66).

For this purpose, we examined the mechanism of effect of a potent competitive inhibitor of the BRD4 BDs-1 and -2, ZL0454, on the intrinsic IIR. ZL0454 binds to the BRD4 BDs-1 and -2 with nanomolar affinity with 30-fold higher specificity for BRD4 over other BET family members (36, 67). In previous studies we have determined the saturating inhibitory dose of ZL0454 for BRD4 inhibition in RSV infection to be 10 μM, a concentration used in subsequent experiments (63). WT, IRF1KD cells were mock or RSV infected in the absence or presence of BRD4i, and expression of the core intrinsic innate response genes were measured.

**Figure 9:**
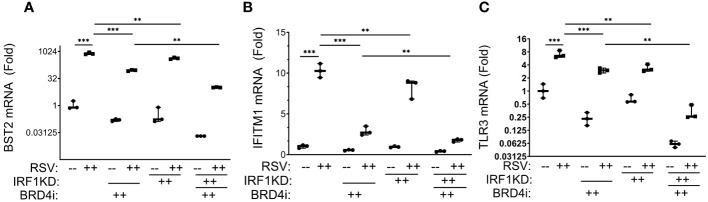
BRD4 BD interactions cooperate with IRF1 in the intrinsic immune response. Wild type (WT) or IRF1 KD hSAECs were treated in the presence or absence of BRD4i (ZL0454, 10 μM). mRNA expression was measured by Q-RT-PCR. Shown is fold change normalized to *PPIA*. **(A)** *BST2*; **(B)** *IFITM1*; **(C)** *TLR3* mRNA ***, P< 0.001; **, P<0.01.

We observed that BRD4i reduced RSV-induced expression of *BST2* mRNA in WT cells vs solvent treated cells from 795 ± 116 fold in RSV-infected to 90 ± 8-fold in BRD4i-treated cells (P<0.001; **Figure 9A**). Similarly, IRF1 KD reduced RSV inducible *BST2* expression by 2-fold, to 425 ± 50-fold (P<0.0001; note that data are plotted on log-transformed scale to demonstrate dynamic changes in expression). Importantly for this analysis, the addition of BRD4i to IRF1 KD further inhibited *BST2* mRNA expression over that produced by BRD4i alone or IRF1 KD alone (**Figure 9A**).

A similar pattern of inhibition was observed for RSV-induced *IFITM1* and *TLR3* mRNA expression, with BRD4i treatment alone producing a greater magnitude of inhibition than that produced by IRF1 KD alone, and the addition of BRD4i in the context of IRF1 KD produces a greater inhibition than either BRD4i alone or IRF1 KD alone (**Figures 9B, C**). These findings suggested that IRF1 and BRD4 work synergistically for the expression of intrinsic IIR genes.

### 3.8 BRD4i disrupts BRD4 pSer2 Pol II complex formation

Our previous XChIP experiments concluded that BRD4 is recruited to innate response genes independently of IRF1. As a component of the PTEF-b complex, BRD4 plays a central role in transcriptional elongation through its ability to potentiate the phosphorylation of RNA Pol II (58). Our previous affinity purification-MS studies of the RSV-induced BRD4 interactome discovered that RSV induced BRD4 binding to two components of the RNA Polymerase complex, POLR2A and POL2RD. Interestingly, these two subunits demonstrated differential sensitivity to BRD4i, with the POL2RA/220 kDa RNA Pol II subunit as being induced into the BRD4 complex in a manner resistant to BRD4i, whereas the POL2RD subunit was disrupted by BRD4i. However, these studies were not designed to identify whether BRD4i interfered with pSer2-modified POL2RA binding to BRD4.

**Figure 10:**
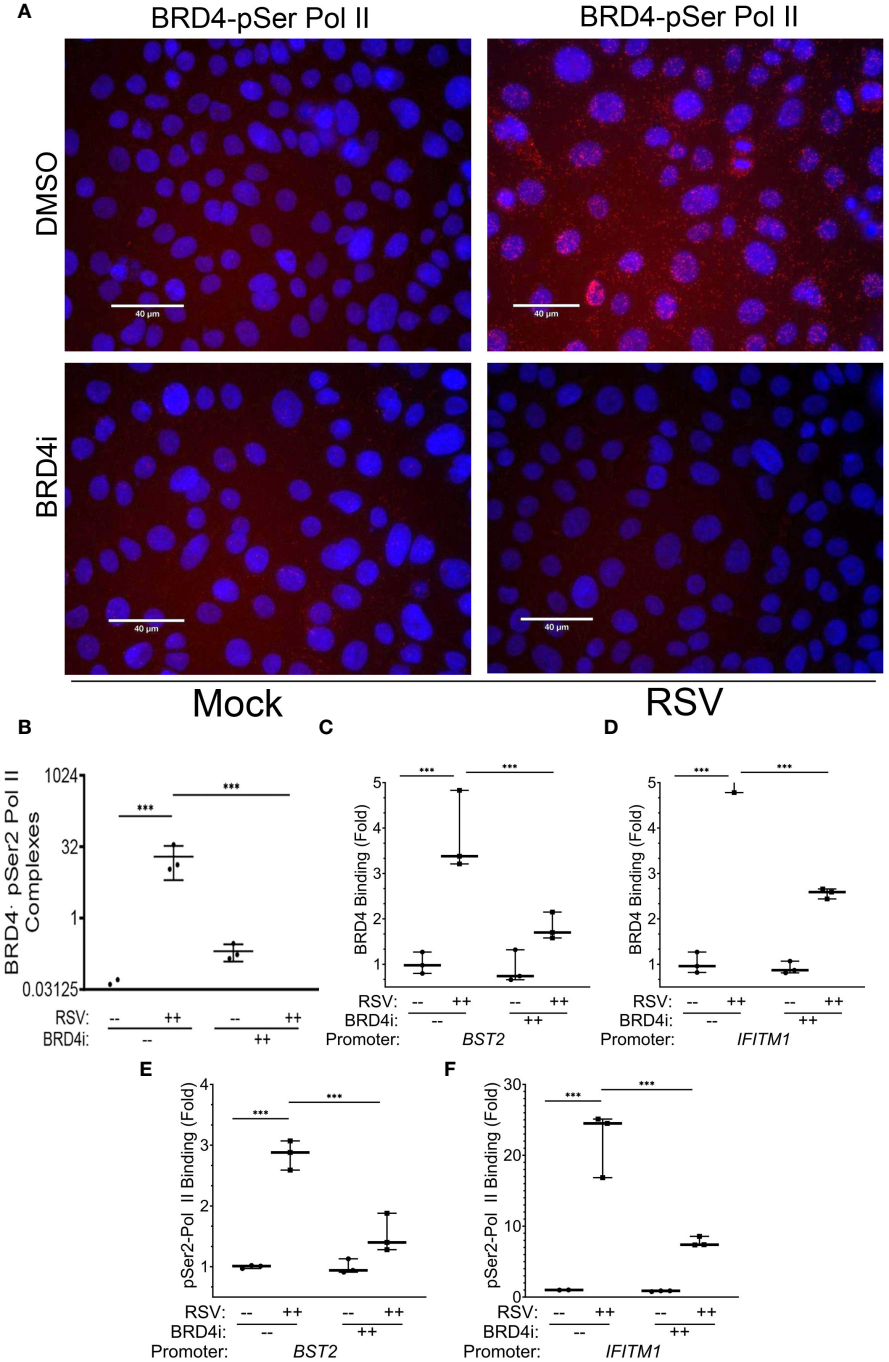
BRD4 BD interactions are required for BRD4 and pSer2 Pol II recruitment. Wild type (WT) hSAECs were treated in the presence or absence of BRD4i (ZL0454, 10 mM). **(A)** PLAs were performed with mouse anti-BRD4 and rabbit anti-pSer2 Pol II Abs. Nuclei are counterstained with DAPI (blue). BRD4·pSer2 Pol II complexes are indicated by red dots. Scale marker is 30 μm. **(B)** Quantitation of BRD4·pSer2 Pol II complexes. BRD4-pSer2 Pol II complexes in n=3 independent fields (>50 nuclei/field) were quantified. ***, P<0.001. XChIP was performed to measure BRD4 binding to **(C)** *BST2* and **(D)** *IFITM1* proximal promoters. Shown are fold enrichment of BRD4 binding by Q-gPCR. **(E, F)** XChIP for pSer2 Pol II binding. Fold enrichment of pSer2 Pol II binding by Q-gPCR. ***, P< 0.001.

To extend these data, we examined the effect of BRD4i on pSer2 Pol II binding to BRD4 using PLA. In mock-infected cells, we were unable to observe significant BRD4·pSer2 Pol II complexes (**Figure 10A**, and quantitation in **Figure 10B**). By contrast, RSV infection induced a 19-fold induction of BRD4 · pSer2 Pol II complexes, with >80% of cells demonstrating BRD4·pSer2 Pol II foci (P=0.03, post-hoc; **Figure 10A**). Strikingly, the association of BRD4 with pSer2 Pol II in RSV infected cells was nearly completely inhibited by BRD4i (19 ± 13-fold vs ND, **Figure 10A**). These data provide a direct mechanism for how BRD4i silences the expression of intrinsic IIR genes by disrupting the formation of- or stability of BRD4·pSer2 Pol II complexes.

### 3.9 BRD4i disrupts BRD4 recruitment to the core intrinsic IIR genes

The mechanisms controlling BRD4 CRC targeting to the intrinsic IIR genes are not understood; using nonselective BET inhibitors, it has been observed that BRD4 binds super enhancers and cell type specification genes through its acetyl-histone binding domain (68). As a separate mechanism, we earlier described that BRD4 is recruited to viral inducible cytokine genes through association with the sequence specific binding activities of the RelA/NFκB transcription factor (31). To provide further understanding for the IRF1-independent effect of BRD4i, we examine the effect of BRD4i on RSV-inducible BRD4 recruitment. For these studies, BRD4 binding to innate response genes was measured in response to RSV in the absence or presence of BRD4i by XChIP.

In response to RSV infection, BRD4 binding to *BST2* increased 3.8 ± 0.9-fold in solvent treated cells, but was reduced by BRD4i to 1.8 ± 0.3-fold (P<0.0001, post-hoc, **Figure 10C**). A similar effect was seen on BRD4 binding to the *IFITM1* promoter, where a 5.8 ± 0.9-fold increase in response to RSV was reduced to 2.6 ± 0.1-fold by BRD4i (P<0.0001, post-hoc; **Figure 10D**).

These reductions in BRD4 binding were mirrored by the reduction in pSer2 Pol II binding to the intrinsic IIR genes. For *BST2*, the RSV-induced 2.8 ± 0.2-fold increase in pSer2 Pol II binding was reduced to 1.5 ± 0.3-fold in the presence of BRD4i (P=0.002, post-hoc; **Figure 10E**). BRD4i reduced the 22.2 ± 4.6-fold increase in pSer2 Pol II binding to *IFITM1* to 7.8 ± 0.7-fold (P=0.0009, post-hoc, **Figure 10F**). These data indicate that the BRD4 BD is required for both BRD4 interaction with pSer2 Pol II and RSV-induced pSer2 Pol II gene recruitment.

### 3.10 BRD4 BD mediates IRF1 interaction with pSer2 Pol II

We were surprised to see the dramatic inhibition of BRD4i pSer2 Pol II binding, since IRF1 is engaged with pSer2 Pol II in the absence of viral infection, and pSer2 Pol II binding to intrinsic IIR genes is IRF1-dependent (**Figure 6**). To potentially explain these observations, we hypothesized that IRF1 interaction with BRD4 was disrupted by the BRD4i, displacing pSer2 Pol II.

**Figure 11:**
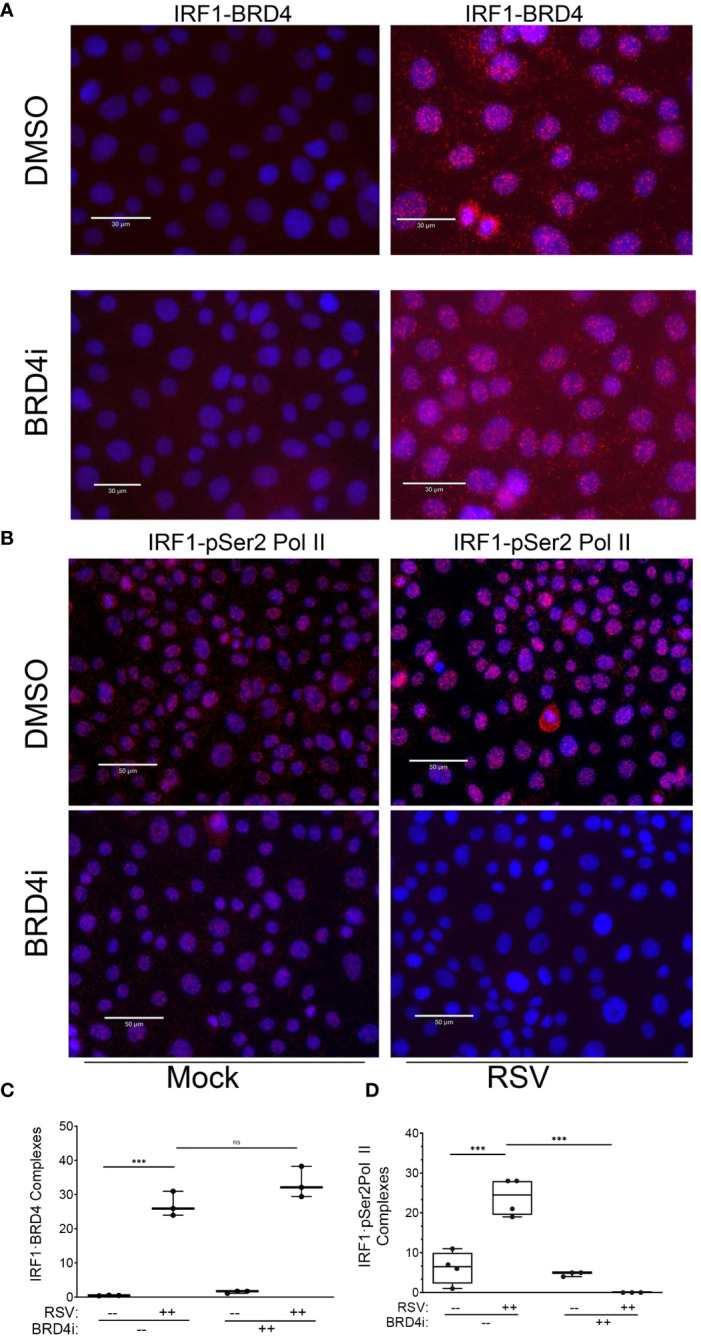
BRD4 BD mediates IRF1 interaction with pSer2 Pol II. Proximity ligation assays were performed with mouse anti-IRF1 and rabbit anti-BRD4 Abs on mock or RSV infected hSAECs (MOI 1, 24 h) in the absence (solvent) or presence of BRD4i. Nuclei are counterstained with DAPI (blue). **(A)** Scale marker is 30 μm. **(B)** Scale marker is 50 μm. **(C)** Quantitation of IRF1·BRD4 complexes. IRF1·BRD4 complex intensity was quantitated in independent fields, normalized to cell count. Each symbol represents an independent high-powered field (n>20 cells/field; 3 fields). **(D)** Quantitation of IRF1·pSer2 Pol II complexes. Complex intensity was quantitated in independent fields, normalized to cell count. Each symbol represents an independent high-powered field (n>20 cells/field; 4 fields). ***, P<0.001; n.s., not significant.

To test this, we examined the effect of BRD4i on BRD4·IRF1 and IRF1·pSer2 Pol II complex formation using PLA. Mock or RSV infected hSAECs were treated with solvent (DMSO) or BRD4i and BRD4·IRF1 complexes quantitated by PLA. We found that the 25 ± 5-fold increase in BRD4·IRF1 complex formation by RSV infection was unaffected by BRD4i (P=n.s.; **Figure 11A** and quantitated in **Figure 11C**). And surprisingly, the BRD4i dramatically reduced the 25 ± 8-fold RSV-induced IRF1-pSer2 Pol II complex to levels less than seen in mock-infected cells (P<0.001, post-hoc; **Figure 11B** and quantitated in **Figure 11D**). These data indicate that RSV induces IRF1 interaction with BRD4 independent of the BD-1 and -2 domains, yet disrupts the RSV induced interactions with pSer2 Pol II.

## 4 Discussion

RSV is a major human pathogen that preferentially infects highly differentiated airway epithelial cells. Here we demonstrate the presence of an intrinsic innate core program in TP63^+^/KRT5^+^/KRT14^+^-expressing airway basal cells capable of differentiating into ciliated and club-cell-like cells. Using next generation sequencing, we observe that these small airway basal cells show a striking conservation of the intrinsic IIR seen in multipotent mesenchymal, neuronal and pancreatic tissue cells. Here we identify a core of the intrinsic IIR as including *BST2, TLR3* and *IFITM1*; these are *direct* IRF1 targets because: 1) their expression is inhibited by IRF1 KD; 2) they are activated by IRF1 expression; and 3) IRF1 binds directly to their promoters in chromatin immunoprecipitation. Moreover, this core of intrinsic IIR genes are in a metastable state under ambient IRF1 control, engaged with activated RNA Pol II and capable of being activated or suppressed by changes in IRF1 expression. In mock-infected cells, we find that pSer2 Pol II is engaged with the core proximal promoters, a hallmark of polymerase processivity. Using *in situ* PLA and XChIP in wild type and IRF1 KD cells, we find that not only does IRF1 complex with pSer2 Pol II in uninfected cells, but stable association of pSer2 Pol II on core promoters is dependent on IRF1 expression. However, IRF1 ectopic expression at levels exceeding those produced by viral replication are insufficient for full activation of the intrinsic IIR genes. Our data indicates that, in response to RSV infection, the synergistic role of BRD4 amplifies the mechanism of activation by transcriptional elongation. Here, BRD4 complexes with IRF1 and loads onto the intrinsic core promoters, where its bromodomain interactions are then required for inducible association of pSer2 Pol II. These findings indicate IRF1 and BRD4 cooperate to regulate the intrinsic core promoters by regulating pSer2 Pol II loading and provide new mechanistic insights into the multiple and cooperative pathways of the IIR.

### 4.1 The intrinsic IIR mediates anti-viral protection

In elegant studies employing *in vitro* differentiation models of pluripotent stem cells, earlier work has shown that the intrinsic innate pathway mediates broad anti-viral protection (25),. Here, pluripotent stem cells are markedly resistant to a variety of RNA and DNA viruses. Interestingly, the intrinsic IIR pathway consists of a subgroup of the spectrum of IFN stimulated genes that lack effects on cell cycle progression. As these cells commit to differentiation programs, the intrinsic IIR is silenced and IFN-inducible ISG become the primary anti-viral defense mechanism. Although previously elucidated for mesenchymal, neuronal and pancreatic tissue cells, the presence and characteristics of intrinsic IIR in airway basal cells is not fully understood (25).

In natural infections and human and animal models, RSV has tropism for differentiated and ciliated epithelium, where it produces inflammation, ciliary dysfunction, necrosis and epithelial sloughing (11, 12, 69, 70). RSV replication in the lower respiratory tract is particularly significant, as this regionalized epithelium is responsible for expression of neutrophilic chemokines, airway remodeling factors and triggering epithelial plasticity (13, 71). In response to viral induced necrosis of the lower airway, TP63+ basal cells undergo epithelial plasticity to repopulate the lower airway and alveolar surfaces (21, 23, 24). Protection of this basal cell population from viral infection is important for the regeneration of the epithelial surface. Previous work in ALI models have shown that basal cells are relatively resistant to RSV infection (69), but become RSV sensitive after physical or chemical disruption of the epithelium (33). In addition, in basal cells, RSV replicates to lower titers than in highly differentiated epithelial cells. These data suggest that both the environmental niche and intrinsic properties of basal cells are mechanisms used to protect basal cells from natural infections.

In our hierarchical clustering studies, we observe that a number of intrinsic IIRs are constitutively expressed in airway basal cells that mediate anti-viral effects. It is well established that deficiency in IFIT cluster expression (25) and/or IRF1 are associated with enhanced replication of RSV, vesicular stomatitis virus and influenza virus in culture systems (72). However, the focus of this study was on the mechanism of IRF1 in regulating intrinsic IIR expression, not the specific mechanisms of the anti-viral mediators.

### 4.2 A core of the intrinsic IIR genes are direct IRF1 targets

We describe here that IRF1 is constitutively expressed in basal cells of the small airway, and extend this finding to demonstrate that its ambient levels are responsible for expression of a core of intrinsic IIR with potent anti-viral activity. Noted earlier, the presence of auto- and feed-forward amplification ensures a robust innate response to pathogen challenge (72, 73), but makes the identification of ISGs directly under IRF1 control difficult. Here we focus our mechanistic studies by using a rigorous criteria to identify *BST2, IFITM1* and *TLR3* as direct IRF1 gene targets. Interestingly our findings suggest that IRF1-dependent genes are under two modes of regulation; one through IRF1 priming and recruitment of pSer2 Pol II in the uninfected state, and the second through the BRD4 CRC recruiting pSer2 Pol II in the setting of RSV infection.

Although the core intrinsic IIR genes have low levels of expression and are engaged with IRF1 in uninfected cells, our time course RNA seq studies demonstrate that these are still highly inducible by RSV infection. However, despite supraphysiological levels of IRF1 expression, IRF1 expression alone does not fully activate intrinsic IIR genes to the same levels produced by RSV infection. These data are consistent with recent work that implicate IRF1 plays a role in priming inducible type III IFNs and ISGs (73, 74). Our earlier work found that IRF1 expression is in a metastable state, whose constitutive and inducible expression is regulated by epigenetic control of enhancer accessibility (30). Our experiments were not designed to examine changes in chromatin topology, but this will be the focus of future studies to understand how the intrinsic IIR is regulated during the epithelial differentiation program. These data suggest the IRF1-intrinsic IIR gene network is undergoing active chromatin remodeling during differentiation programs.

### 4.3 IRF1 complexes with activated Pol II in uninfected cells

We extend understanding of intrinsic IIR in the airway by demonstrating that constitutive expression the core anti-viral program is dependent on ambient IRF1 levels. Our studies indicate that a fraction of IRF1 is nuclear and in a form engaged with IIR promoters. In embryonic fibroblasts, co-immunoprecipitation experiments demonstrate that IRF1 binds to Pol II (75). However, whether RNA Pol II is in the activated form was not examined. Here we extend this understanding to demonstrate that IRF1 binds to the transcriptionally elongation competent-pSer2 Pol II. Using phospho-specific antibodies in PLA, we are able to demonstrate that IRF1 binds to RNA Pol II in multi-potent airway epithelial cells. That IRF1·pSer2 Pol II complex is functional is supported by our observations that inhibition of IRF1 reduces pSer2 Pol II binding as well as expression of the core intrinsic IIR genes.

Constitutive IRF1 activity has been implicated in intrinsic IIR hepatitis A infection in hepatocytes (74). However, this study did not investigate differences in the intrinsic IIR between stem cells differentiated hepatocytes. Our work builds on and extends this earlier work by placing basal IRF1 expression in controlling intrinsic IIRs in multi-potent stem cells of the airway epithelium. The understanding of how intrinsic IIR regulated RSV replication in the lower airway is incomplete, yet understanding this coordinated response is important because the initial magnitude of replication determines disease severity, inflammation and mucus production in human challenges (7).

### 4.4 Inducible IRF1 cooperates with BRD4 for a high level ISG response

Despite its ambient levels of expression in uninfected cells, IRF1 is induced ~3-fold by RSV replication. In earlier studies we showed that IRF1 is activated by an 5’ upstream enhancer binding NFκB (30) cooperating with the spliced X Box binding protein (XBP1s) (76). That this enhancer was functional was demonstrated using site-specific targeting of a potent KRAB repressor domain to the IRF1 enhancer, where we found suppression of RSV-inducible IRF1 expression and downregulation of type III IFNs, also direct IRF1 targets (76). This work has elucidated IRF1 is a signal integrator in innate response, through a complex 5’ enhancer sequence regulated RSV (76). Our earlier findings that topology of this enhancer sequence is affected by changes in epithelial cell-state may provide insights into how the intrinsic IIR is silenced by differentiation state.

Interpreted collectively, our data suggest that IRF1 may function in two modes- one controlling ambient intrinsic IIR genes through direct basal activation and the second cooperating with the BRD4 CRC in the induction of ISGs in response to viral challenge. There is substantial evidence that activated IRF1 activates genes through cooperative recruitment of RNA Pol II in mechanistically distinct ways. For example, others have shown that IRF1 activates the IFNγ-inducible Guanylate Binding Protein 2 (*gbp2*) gene cooperatively with STAT1. In this study, each transcription factor independently participates in Pol II recruitment (75). In a parallel mechanism, we propose here that IRF1 complexes with- and functionally cooperates with- the BRD4 CRC in activation of intrinsic IIR genes. Although BRD4 binding does not require IRF1, both BRD4 and IRF1 contribute to the recruitment of RNA Pol II, because inhibition of either IRF1 expression or disruption of BRD4 BD reduces pSer2 Pol II interaction with intrinsic response genes.

### 4.5 The BRD4 CRC plays multiple roles in highly inducible gene expression

In differentiated cells, the BRD4 CRC is constitutively engaged with cell-type specification genes and super enhancers across the genome. In response to RSV infection, the BRD4 CRC is recruited to the intrinsic IIR and epithelial plasticity genes. RSV infection increases ~100 proteins to bind the BRD4 complex (64, 77). Through these associated proteins, BRD4 plays multiple functional roles in the coordinate genome innate response, including transcriptional elongation, stimulating enhancer remodeling, and mediating alternative mRNA splicing (65).

In the context of RSV infection, we have shown that BRD4 mediates immediate-early cytokine gene expression through its atypical acetyltransferase- and RNA polymerase -directed kinase activities that promote transcriptional elongation (56–58). Transcriptional elongation is a regulated step in ISG expression mediated by pSer2 Pol II formation, inducing rapid, processive, gene expression (29, 31, 32, 45, 47–53). BRD4 binds the PTEFb-CDK complex that cooperatively phosphorylates the RNA Pol II CTD. Our earlier affinity purification (AP)-MS studies demonstrated that BRD4 also interacts with RNA Pol II subunits, POL2RA and POLR2D (64). Our PLA studies here extend these findings to show BRD4 binds to the activated pSer2 Pol II form, consistent with its role in mediating transcriptional elongation. Finally, the atypical acetyltransferase activity of BRD4 further promotes transcriptional elongation by acetylating histones within the core nucleosome, destabilizing their binding on gene bodies, further promoting Pol II translocation (57).

Other actions of the BRD4 complex are mediated through interactions with the enhancer binding complexes, Med and AP2, enabling enhancer coupling to proximal promoters for gene activation. The roles of enhancer coupling in the intrinsic IIR are not fully understood, although we have found that BRD4 is involved in autoregulatory and amplification loops of many of these enhancer binding proteins.

In a distinct mechanism, we have recently demonstrated that BRD4’s interaction with spliceosome promotes alternative mRNA splicing of a subset of IIR genes (65). One of these pathways controlled by BRD4 alternative splicing is the unfolded protein response, a critical pathway in the intrinsic IIR and metabolic adaptations to RSV infection (34, 76). These studies indicate that BRD4 complex play multiple roles in activating enhancer-dependent, transcriptional elongation and RNA processing of target genes. Whether intrinsic innate genes are alternatively spliced was not addressed in this study and will require further investigation.

### 4.6 The BRD4·IRF1 interaction is enhanced in response to RSV

Here we extend the understanding of a functional protein interaction between BRD4 and IRF1 using a highly sensitive PLA. PLAs have the advantage in preserving protein-protein interactions *in situ* that may not survive a conventional immunoprecipitation enrichment experiment, and because of the distance constraints of cross-linking for PLA signal (e.g., less than 10 Å), suggest that the IRF1 and BRD4 proteins are directly interacting. IRF1 recruitment into the BRD4 CRC was not observed in this earlier study, probably due to the lability of the interaction or stochastic nature of MS sample acquisition. Our findings extend the understanding that IRF1 weakly binds BRD4 constitutively, but robustly interacts with BRD4 in RSV infection. BRD4 protein interactions are mediated by diverse mechanisms including acetylated lysine recognition by the bromodomain and independent interactions through the COOH Terminal Bromo and Extraterminal (BET) domain. The mechanisms driving IRF1 binding to BRD4 complex has not been systematically studied. Because of its resistance to BRD4i, we doubt that this interaction is driven by acetylated lysine modifications of IRF1. More work will be required to identify the domains of BRD4 interacting with IRF1.

### 4.7 BRD4 recruitment to intrinsic response genes is BD-dependent

The mechanisms controlling BRD4 recruitment to target genes are not fully understood. Although BRD4 interacts with acetylated Histone H3 and H4 side chains (57) to bind to superenhancers and cell type specification genes (78), BRD4 CRC is a dynamically restructured upon RSV infection. In the setting of innate activation, BRD4’s association with sequence specific transcription factors, such as RELA/NFκB (78) and AP1 (64) may provide independent mechanisms for targeting the BRD4 CRC to inducible ISGs. Our observations that the BRD4 CRC is recruited to intrinsic innate response genes in response to RSV reproduces earlier studies (31).

An important mode of inducible protein interaction is mediated by acetylated lysine-modified transcription factor binding to the BRD4 bromodomain (BD). Acetylated protein interactions enable dynamic reconfiguration of the BRD4 CRC in response to extracellular signals, differentiation states and viral infections (66). For example, the innate responsive NF-κB/RELA transcription factor is induced by RSV by K310 acetylation that is recognized through both BDs. Here we use a highly selective BRD4i to probe the interactions between IRF1, pSer2 Pol II and BRD4. This BRD4i interacts with both BDs of BRD4 with nM affinity and displaces acetylated histones from the complex (62). Interestingly, we find that BRD4i disrupts the interaction of IRF1 with pSer2 Pol II without affecting the interaction of IRF1 with BRD4. This suggests that BRD4i may disrupt chromatin complexes during evolution of the intrinsic IIR.

Our work extends this acetyl-lysine targeting mechanism by demonstrating BRD4 binding is BD-dependent and IRF1 independent, as RSV-induced BRD4 binding is inhibited by BRD4i and yet occurs effectively in IRF1 KD cells. However, based on the complexity of BRD4-protein interactions, multiple modes of BRD4 targeting to chromatin may exist in other cell types and in response to distinct stimuli.

## 5 Conclusions

The mechanisms of innate protection of the airway basal cell are critical for repopulating the airway after RSV infection, but incompletely understood. Using a model lower airway basal cell, we observe that the basal cell exhibits a highly conserved intrinsic IIR. Here, ambient levels of IRF1 is constitutively complexed with pSer2 Pol II on intrinsic innate IIR genes maintaining them in a ‘primed’ state for activation. Upon viral infection, IRF1 are induced, complexes with BRD4 and both factors load pSer2 Pol II onto target gene promoters. Using small molecule antagonist of the BRD4 BD, we observe that IRF1 interacts with BRD4 independently of the BD, yet stable association of pSer2 Pol II requires both BRD4 and IRF1. These data indicate the coordination between IRF1 and the BRD4 CRC in regulating intrinsic immunity.

## Data availability statement

The datasets presented in this study can be found in online repositories. The names of the repository/repositories and accession number(s) can be found below: https://www.ncbi.nlm.nih.gov/geo GSE179353; and https://www.ncbi.nlm.nih.gov/geo GSE161849.

## Ethics statement

Ethical approval was not required for the studies on humans in accordance with the local legislation and institutional requirements because only commercially available established cell lines were used. Ethical approval was not required for the studies on animals in accordance with the local legislation and institutional requirements because only commercially available established cell lines were used.

## Author contributions

XX: Conceptualization, Data curation, Formal analysis, Investigation, Methodology, Writing – original draft, Writing – review & editing. DQ: Investigation, Methodology, Writing – original draft, Writing – review & editing. AB: Investigation, Methodology, Writing – original draft, Writing – review & editing, Conceptualization, Data curation, Formal analysis, Funding acquisition, Resources, Supervision, Visualization.
